# Comparison of Long-Term Survival and Toxicity of Cisplatin Delivered Weekly versus Every Three Weeks Concurrently with Intensity-Modulated Radiotherapy in Nasopharyngeal Carcinoma

**DOI:** 10.1371/journal.pone.0110765

**Published:** 2014-10-16

**Authors:** Chang-Juan Tao, Li Lin, Guan-Qun Zhou, Ling-Long Tang, Lei Chen, Yan-Ping Mao, Mu-Sheng Zeng, Tie-Bang Kang, Wei-Hua Jia, Jian-Yong Shao, Hai-Qiang Mai, Ai-Hua Lin, Jun Ma, Ying Sun

**Affiliations:** 1 Department of Radiation Oncology, Sun Yat-sen University Cancer Center, State Key Laboratory of Oncology in South China, Collaborative Innovation Center for Cancer Medicine, Guangzhou, Guangdong Province, People's Republic of China; 2 Department of Radiation Oncology, Zhejiang Cancer Hospital, Hangzhou, Zhejiang Province, People's Republic of China; 3 Department of Experimental Research, Sun Yat-sen University Cancer Center, State Key Laboratory of Oncology in South China, Collaborative Innovation Center for Cancer Medicine, Guangzhou, Guangdong Province, People's Republic of China; 4 Department of Pathology, Sun Yat-sen University Cancer Center, State Key Laboratory of Oncology in South China, Collaborative Innovation Center for Cancer Medicine, Guangzhou, Guangdong Province, People's Republic of China; 5 Department of Nasopharyngeal Carcinoma, Sun Yat-sen University Cancer Center, State Key Laboratory of Oncology in South China, Collaborative Innovation Center for Cancer Medicine, Guangzhou, Guangdong Province, People's Republic of China; 6 Department of Medical Statistics and Epidemiology, School of Public Health, Sun Yat-sen University, Guangzhou, Guangdong Province, People's Republic of China; University of Nebraska Medical Center, United States of America

## Abstract

**Background:**

We aimed to compare the long-term survival outcomes and acute toxicity of cisplatin administered weekly versus every three weeks concurrently with intensity-modulated radiotherapy (IMRT) in patients with nasopharyngeal carcinoma (NPC).

**Methods:**

This was a retrospective review of 154 patients with histologically proven, non-disseminated NPC who were treated using IMRT between January 2003 and December 2007. Seventy-three patients (47.4%) received 5–7 weeks of 30–40 mg/m^2^ cisplatin weekly; 81 patients (52.6%) received two or three cycles of 80 mg/m^2^ cisplatin every three weeks. IMRT was delivered at 68 Gy/30 fractions to the nasopharyngeal gross target volume and 60–66 Gy to the involved neck area.

**Results:**

The clinical characteristics and treatment factors of the two groups were well-balanced. The median follow-up was 74 months (range, 6–123 months), and the 5-year overall survival, disease-free survival, locoregional relapse-free survival, and distant metastasis–free survival rates were 85.2% vs. 78.9% (*P* = 0.318), 71.6% vs. 71.0% (*P* = 0.847), 93.5% vs. 92.6% (*P* = 0.904), and 80.9% vs. 80.1% (*P* = 0.925) for the group treated every three weeks and weekly, respectively. Subgroup analyses indicated no significant differences in the survival rates of the two groups among patients with early- or advanced-stage disease. The incidence of acute toxicities was similar between groups.

**Conclusion:**

IMRT with concurrent cisplatin administered weekly or every three weeks leads to similar long-term survival outcomes and acute toxicity in NPC regardless of whether patients have early- or advanced-stage disease.

## Introduction

Nasopharyngeal carcinoma (NPC) is highly prevalent in southern China; incidence rates range between 15 and 50 per 100,000 individuals [1]. Due to the anatomical location and radiosensitivity of NPC, radiation therapy (RT) is the mainstay treatment. According to the 7th Edition of the American Joint Commission on Cancer (AJCC) staging system [2], 60–70% of patients present with stage III–IVB disease at diagnosis [3]. Several randomized clinical trials and meta-analyses have consistently demonstrated that concurrent chemoradiotherapy (CCRT) confers survival benefit in locoregionally advanced NPC [4]–[11].

The National Comprehensive Cancer Network recommends that CCRT with cisplatin (CDDP) be delivered at an intermediate dose weekly or at a high dose at 3-week intervals for stage II–IVB NPC. The Intergroup 0099 trial (INT-0099) [3] and NPC-9901 trial [5] compared concurrent CDDP (100 mg/m^2^) every three weeks with RT alone; compliance to the intensive regimen was poor, and CCRT was associated with a high incidence of acute toxicity. Two other phase III clinical trials [6]–[7] evaluated the efficiency of CCRT with weekly CDDP (40 mg/m^2^) during RT; CCRT conferred survival benefit and weekly CDDP was well-tolerated. Therefore, it is of great importance to identify the optimal administration schedule for CDDP during CCRT in patients with NPC.

To the best of our knowledge, only one study [12] has compared the dose delivery and survival outcomes of weekly CDDP versus CDDP every three weeks delivered concurrently with RT in locally advanced NPC. However, in that study, the clinical characteristics and treatment factors were unevenly distributed, and the majority of patients were treated with 3-dimensional conformal RT. Radiation technology has evolved rapidly in recent years, especially with the introduction of intensity-modulated radiotherapy (IMRT). In NPC, this treatment modality has excellent local control and less late toxicities [13]. With the widespread application of IMRT, reevaluating the survival outcomes and toxicity of concurrent weekly CDDP versus CDDP every three weeks in patients with NPC undergoing CCRT would be worthwhile.

Therefore, we aimed to compare the long-term survival outcomes and acute toxicities of weekly CDDP versus CDDP every three weeks delivered concurrently with IMRT in patients with NPC. Our results will help guide clinical CCRT treatment strategies in NPC.

## Materials and Methods

### Patient characteristics

Between January 2003 and December 2007, we reviewed the records of 749 patients with newly diagnosed, untreated, non-disseminated NPC who were treated using IMRT at our center. We excluded 595 patients (79.4%): 214 (28.6%) who were treated with RT only, 274 (36.6%) who received neoadjuvant chemotherapy, 86 (11.5%) who received concurrent chemotherapy using a combination of CDDP and 5-fluorouracil, and 21 (2.8%) who received concurrent chemotherapy with taxol. We included the remaining 154 patients in our retrospective study; 115 were male and 39 were female (male:female ratio, 2.95∶1) and the median age was 44.0 years (range, 13–73 years). One hundred and fifty patients (97.4%) had a pathological diagnosis of World Health Organization (WHO) type II or III NPC, only one patient (0.6%) had WHO type I NPC, and three patients (1.9%) had basaloid squamous cell carcinoma. The ethics committee of Sun Yat-sen University Cancer Center approved this study. Written consent was waived because this was a retrospective study; verbal consent was obtained from the patients via telephone and documented in the informed consent form if the patient agreed to participate in this study, as was consent on behalf of the children enrolled. The Institutional Review Board approved the use of verbal consent.

All patients underwent pre-treatment evaluation, which included complete patient history, physical and neurological examination, hematological and biochemical profiles, magnetic resonance imaging (MRI) scan of the neck and nasopharynx, chest radiography, abdominal sonography, and single-photon emission computed tomography whole-body bone scans. Positron emission tomography/computed tomography (CT) was performed on 41 patients (26.6%). All patients were restaged according to the 7th edition of the AJCC staging system. The stage distribution was as follows: stage II, 40/154 (26%); stage III, 66/154 (42.8%); stage IVA, 38/154 (24.7%); stage IVB, 10/154 (6.5%). Table 1 lists the characteristics of the patient cohort.

**Table 1:** Clinical characteristics of patients.

Characteristic	Weekly cisplatin (n = 73)	Cisplatin every three weeks (n = 81)	*P* *
Age (years)			.351
≤50	52	63	
>50	21	18	
≤18	0	3	.247
>18	73	78	
Sex			.581
Male	56	59	
Female	17	22	
Histology			.116
WHO I	1	0	
WHO II	7	3	
WHO III	65	75	
BSCC	0	3	
T classification†			.651
T1	7	13	
T2	17	17	
T3	31	30	
T4	18	21	
N classification†			.538
N0	14	13	
N1	42	55	
N2	12	8	
N3	5	5	
Staging†			.939
II	18	22	
III	33	33	
IVA	17	21	
IVB	5	5	
OTT (days)			.554
Median	43	44	
SD	4.3	4.0	
Response rate			.258
CR	60	61	
PR	11	19	

WHO: World Health Organization; BSCC: basaloid squamous cell carcinoma; CR: complete response; PR: partial response; SD: standard deviation; OTT: overall treatment time.

*P-values were calculated using the chi-square test (or Fisher's exact test) and Mann–Whitney U test.

† According to the 7th AJCC/International Union against Cancer staging system.

### Radiotherapy

All patients were immobilized in the supine position with a head, neck, and shoulder thermoplastic mask. We obtained two sets of images, i.e., with and without contrast, from the CT simulator for treatment planning purposes. CT was performed after administering intravenous contrast medium, and we obtained 3-mm slices from the head to 2 cm below the sternoclavicular joint. The primary tumor and upper neck above the caudal edge of the cricoid cartilage were treated by IMRT. The target volumes were delineated using a previously described institutional treatment protocol [14] in accordance with the International Commission on Radiation Units and Measurements reports 50 and 62. The clinical target volumes (CTVs) were individually delineated based on the tumor invasion pattern [15]. The contoured images were transferred to a Corvus version 3.0 inverse IMRT planning system (Peacock; Nomos Corp., Deer Park, IL, USA). The prescribed radiation dose (as per the protocol) was total dose of 68 Gy in 30 fractions at 2.27 Gy/fraction to the planning target volume (PTV) of the primary gross tumor volume (GTV), 60–64 Gy to the nodal GTV PTV, 60 Gy to the CTV-1 PTV (i.e., high-risk regions), and 54 Gy to the CTV-2 PTV (i.e., low-risk regions) and CTV-N (i.e., neck nodal regions). Treatment was delivered by a dynamic, multileaf intensity-modulating collimator (MIMiC; Nomos Corp., Sewickley, PA, USA). A conventional anterior or anteroposterior opposing cervical technique was used for the lower neck. All patients were treated with one fraction daily over five days per week. All targets were treated simultaneously using the simultaneous integrated boost technique.

### Concurrent chemotherapy

All patients were treated with CCRT; eight patients (5.2%) also received adjuvant chemotherapy using CDDP and 5-fluorouracil for two or three cycles. CCRT was initiated on the first day of RT and the choice of every three weeks or weekly CDDP was based on oncologists' opinions. Seventy-three patients (47.4%) received 30–40 mg/m^2^ CDDP weekly for 5–7 planned weeks, and 81 patients (52.6%) received two or three cycles of 80 mg/m^2^ CDDP every three weeks. In both groups, 5-hydroxytryptamine 3 antagonists and dexamethasone were administered as antiemetic prophylaxis.

### Follow-up

The duration of follow-up was calculated from the first day of treatment to either the day of death or day of the last follow-up. Patients were examined at least every three months during the first two years, and every six months thereafter until death. At every follow-up, we assessed disease status with complete physical examination, nasopharyngoscopy, hematological and biochemical profiles, chest radiography, abdominal ultrasonography, and CT/MRI scans of the nasopharynx and cervical region. Acute toxicities were scored according to the Common Terminology Criteria for Adverse Events v3.0 (CTCAE v3.0).

### Statistical analysis

We used Statistical Package for the Social Sciences version 16.0 (SPSS, Chicago, IL, USA) for statistical analysis. We compared and analyzed the clinical characteristics and toxicity rates of the two treatment groups using the chi-square test (or Fisher's exact test, if indicated). Overall treatment time and cumulative CDDP dose were compared using the Mann–Whitney U test. All events were measured from the start of treatment. The following end points (time to the first defined event) were assessed: overall survival (OS), disease-free survival (DFS), locoregional relapse–free survival (LRRFS), distant metastasis–free survival (DMFS). Actuarial rates were calculated using the Kaplan–Meier method [16]. Multivariate analyses using the Cox proportional hazards model were used to test for independent significance using backward elimination of insignificant explanatory variables of different parameters [17]. Host factors (age and sex) were included as covariates in all tests. The criterion for statistical significance was set at *a* = 0.05 and *P*-values were determined using 2-sided tests.

## Results

### Patient characteristics

Both groups were well-balanced in terms of baseline demographic and clinical characteristics and treatment factors (Table 1). Tumor response was evaluated via endoscopy and MRI scans of the neck and nasopharynx at three months after RT. The composite overall response rate for the primary site and neck region was 97.3% and 98.7% for the weekly and every three week CDDP group, respectively (*P* = 0.258).

### Treatment compliance and acute toxicities

All 154 patients tolerated the treatment well and completed the prescribed dose of RT. The median overall treatment time was 43 days in the weekly group and 44 days in the every three weeks group (*P* = 0.554). In the weekly group, 66 patients (90.4%) received at least five weeks of CDDP and only four patients (5.5%) received seven weeks of CDDP. In the every three weeks group, 72 patients (88.9%) completed two cycles of CDDP and five patients (6.2%) received three cycles of CDDP. The median cumulative dose received during concurrent CCRT was 180 and 160 mg/m^2^ CDDP for the weekly and every three weeks group, respectively (*P* = 0.10). Reductions in the number of chemotherapy cycles were mostly due to patient refusal, severe mucositis, or prolonged severe leucopenia.

Systemic toxicities were similar in both groups (Table 2). No treatment-related deaths were observed in either cohort. The most commonly recorded non-hematological adverse event was grade 3–4 mucositis in 23 patients (31.5%) in the weekly group and 24 patients (29.6%) in the every three weeks group (*P* = 0.139). The most common hematological adverse event was grade 3–4 leucopenia in six patients (8.2%) in the weekly group and five patients (6.2%) in the every three weeks group (*P* = 0.312).

**Table 2:** Acute toxicity according to CTCAE v3.0.

Toxicity	Weekly cisplatin (n = 73)					Every three week cisplatin (n = 81)					*P* *
	Grade0	Grade1	Grade2	Grade3	Grade4	Grade0	Grade1	Grade2	Grade3	Grade4	
Hematological											
Leucopenia	30	17	20	5	1	23	28	25	5	0	0.312
Anemia	58	9	4	2	0	61	17	3	0	0	0.233
Thrombocytopenia	62	4	2	3	2	74	2	2	2	1	0.583
Non-hematological											
Dermatitis	0	59	13	1	0	0	54	24	3	0	0.121
Mucositis	0	29	21	23	0	0	23	34	23	1	0.139
Dysphagia	29	33	8	3	0	31	32	13	5	0	0.715
Nausea/Vomiting	32	24	17	0	0	36	27	15	3	0	0.295
Xerostomia	15	27	31	0	0	11	31	39	0	0	0.498
Ototoxicity	52	19	2	0	0	57	23	1	0	0	0.767
Neurotoxicity	70	2	1	0	0	77	3	1	0	0	0.632

*P-values were calculated with the chi-square test (or Fisher's exact test, if indicated).

### Patterns of failure and survival rates

The overall median follow-up time was 74 months (range, 6–123 months). Twelve of the 154 patients (7.8%) developed locoregional relapse. There was distant metastasis in 29/154 patients (18.8%), and 35/154 patients (22.7%) died. The sites of relapse were local in 7/154 patients (4.5%), regional in 7/154 patients (4.5%), and both local and regional in 2/154 patients (1.3%). Three patients (1.9%) experienced both locoregional and distant failure.

**Figure 1:**
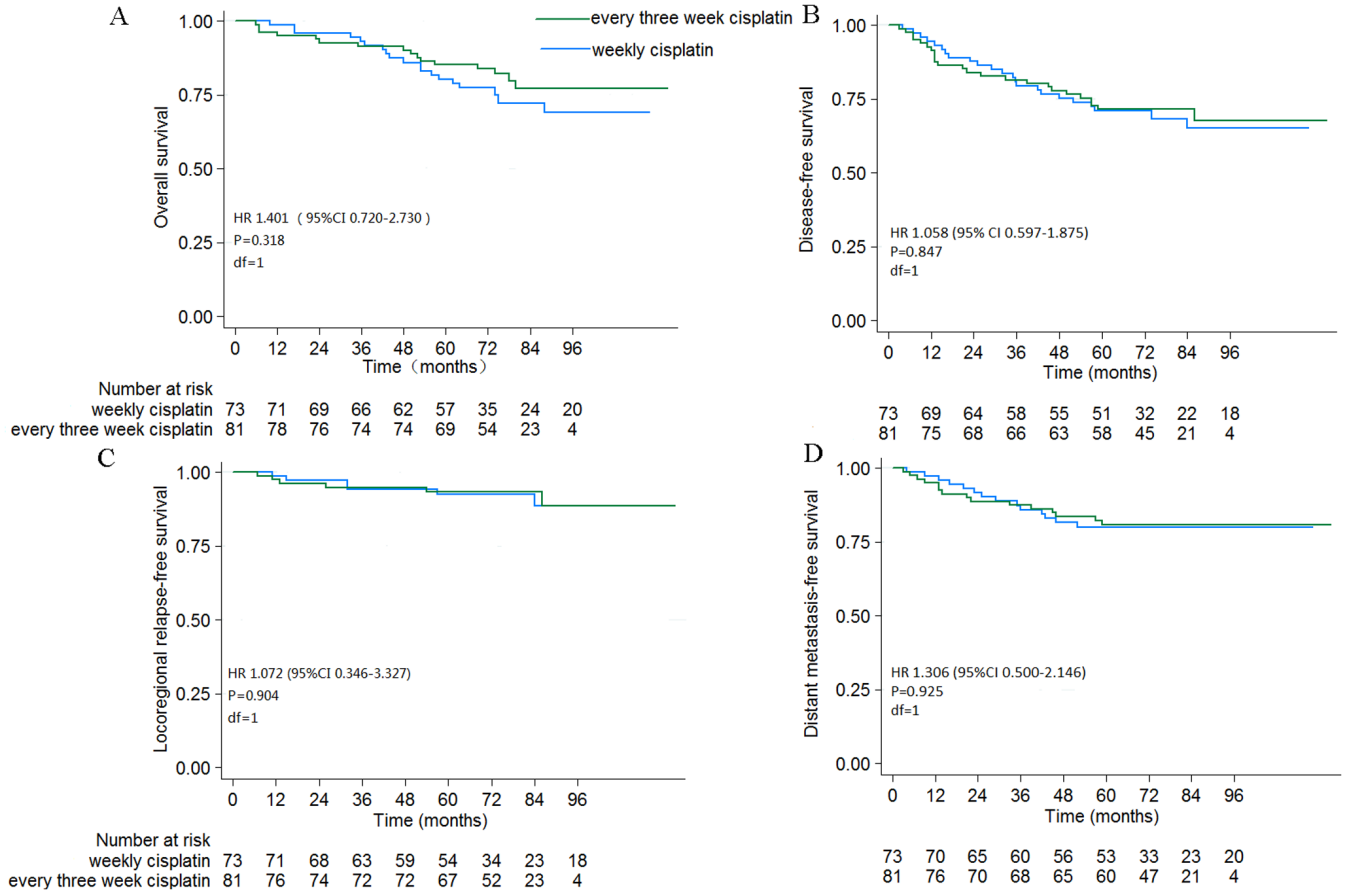
Survival curves for patients with NPC. (A) OS, (B) DFS, (C) LRRFS, and (D) DMFS. Hazard ratios (HRs) were calculated with an unadjusted Cox proportional hazards model; *P*-values were calculated with the unadjusted log-rank test.

**Figure 2:**
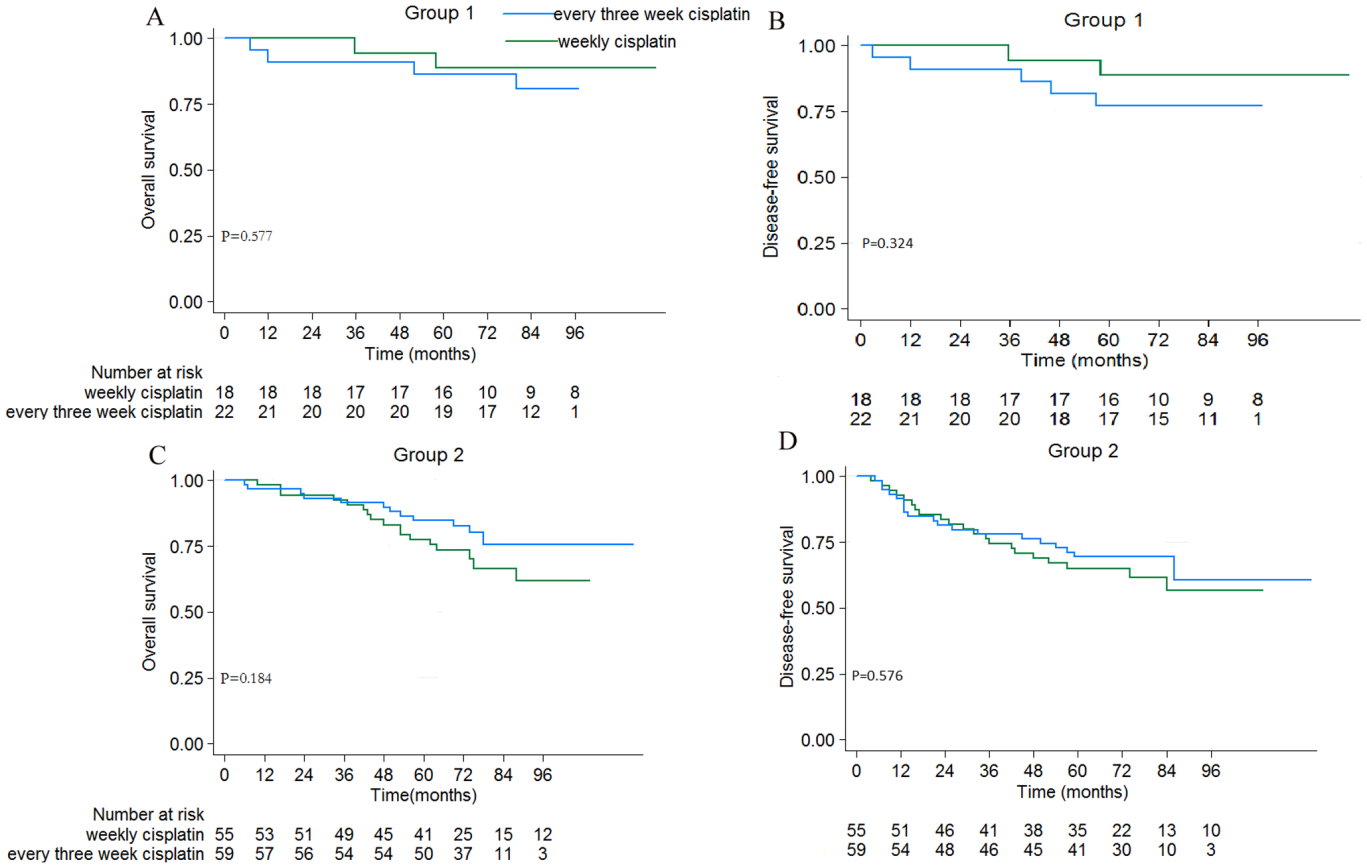
OS and DFS curves for NPC patients stratified by clinical stage. Group 1: patients with stage II disease; Group 2: patients with stage III–IVB disease. *P*-values were calculated with the unadjusted log-rank test.

The overall 5-year OS, DFS, LRRFS, and DMFS rates were 82.3%, 71.3%, 93.1%, and 80.5%, respectively. The 5-year OS, DFS, LRRFS, and DMFS rates were not significantly different between the two treatment groups (every three weeks vs. weekly: OS = 85.2% vs. 78.9%, *P* = 0.318; DFS = 71.6% vs. 71.0%, *P* = 0.847; LRRFS = 93.5% vs. 92.6%, *P* = 0.904; DMFS = 80.9% vs. 80.1%, *P* = 0.925; Fig. 1). The 5-year OS and DFS rates for patients with early- or advanced-stage disease in the two treatment groups were not significantly different (Fig. 2).

### Prognostic factors

Univariate analysis revealed that age and N classification were significant prognostic factors for OS (*P* = 0.009 and 0.011, respectively). The 5-year OS rate for patients who received cumulative CDDP≥160 mg/m^2^ was 84.1% compared to 73.3% for patients who received cumulative CDDP<160 mg/m^2^ (*P* = 0.175).

Multivariate analysis was performed to adjust for various prognostic factors. The following parameters were included in the Cox proportional hazards model by backward elimination of insignificant explanatory variables: age (>50 years vs. ≤50 years), sex, T classification (T1–2 vs. T3–4), N classification (N0–1 vs. N2–3), concurrent chemotherapy regimen (CDDP weekly vs. every three weeks), and cumulative CDDP dose (≥160 mg/m^2^ vs. <160 mg/m^2^). Multivariate analysis demonstrated that the CCRT regimen was not a significant prognostic factor for any end point. Age and N classification were significant predictive factors for OS; furthermore, N classification was the only significant prognostic factor for DFS and DMFS (Table 3).

**Table 3:** Summary of multivariate analysis of prognostic factors in the 154 patients.

End point	HR (95% CI)	*P*-value†
**OS**		
Age,>50 years vs. ≤50 years	2.809 (1.404–5.620)	0.004
N classification (N0–1 vs. N2–3)	2.830 (1.388–5.767)	0.004
Chemotherapy regimen, weekly vs. every three weeks	0.862 (0.428–1.738)	0.679
**DFS**		
N classification (N0–1 vs. N2–3)	2.241 (1.213–4.141)	0.010
Chemotherapy regimen, weekly vs. every three weeks	1.099 (0.604–2.001)	0.757
**DMFS**		
N classification (N0–1 vs. N2–3)	2.752 (1.271–5.961)	0.010
Chemotherapy regimen, weekly vs. every three weeks	1.331 (0.607–2.917)	0.475
**LRRFS**		
Chemotherapy regimen, weekly vs. every three weeks	1.280 (0.382–4.288)	0.689

HR: hazard ratio; CI: confidence interval; OS: overall survival; DFS: disease-free survival; DMFS: distant metastasis–free survival; LRRFS: locoregional relapse–free survival.

† P-values were calculated using an adjusted Cox proportional hazards regression model adjusted for age (>50 years vs. ≤50 years), sex (male vs. female), T classification (T1–2 vs. T3–4), N classification (N0–1 vs. N2–3), chemotherapy regimen, (weekly vs. every three weeks) and cumulative cisplatin dose, (≥160 mg/m2 vs. <160 mg/m2). Only variables that were significantly associated with survival are presented except for the chemotherapy regimen.

## Discussion

NPC is a highly chemosensitive tumor and CCRT has long been a standard treatment option for locoregionally advanced NPC. Treatment regimens delivering medium doses of CDDP weekly or high CDDP doses every three weeks have been evaluated. Weekly medium doses are efficient and simple to administer, especially for outpatients; high doses every three weeks are more effective for reducing distant metastasis, but may lead to more severe acute toxicities [4]–[7].

In this retrospective study, we observed that CDDP-based (both weekly and every three weeks) CCRT led to similar long-term survival outcomes and acute toxicities in patients with NPC receiving IMRT regardless of whether the patients had early- or advanced-stage disease. Our results are in agreement with that of Jagdis et al. [12], which showed that 40 mg/m^2^ CDDP weekly and 100 mg/m^2^ CDDP every three weeks had similar deliverability, toxicity profiles, and comparable survival outcomes in locally advanced NPC.

In our study, the overall 5-year OS, DFS, LRRFS, and DMFS rates were 82.3%, 71.3%, 93.1%, and 80.5%, respectively. CDDP delivered both weekly or every three weeks concurrently with IMRT achieved excellent OS. Distant metastasis was the predominant mode of failure. One approach to improving the strategy tested in this study would be changing the sequence to induction–concurrent, as substantially better tolerance and compliance rates have been reported for induction–concurrent regimens [18]. The meta-analysis by OuYang et al. in 2013 [19] also showed that neoadjuvant chemotherapy effectively enhances OS and reduces the rate of distant metastasis in NPC.

With respect to dose delivery, both treatment groups achieved similar cumulative dose intensities; however, a low proportion of patients completed seven weeks of weekly CDDP or three cycles of every three week CDDP. The compliance rates in the present study are lower than that of previously published trials [4]–[7]. This may be because the weekly CDDP regimen was mostly administered to outpatients and the regimen given every three weeks was mainly administered to inpatients at our center. Poor outpatient compliance and inadequate numbers of inpatient beds led to delays in administering chemotherapy, which often resulted in patients missing the final cycle of chemotherapy.

We adopted an every three week CDDP dose of 80 mg/m^2^ in our study, which was effective with low toxicity. The INT-0099 regimen is widely used, where 100 mg/m^2^ CDDP is administered on days 1, 22, and 43 with radical RT, given its impressive results compared to RT alone when treating NPC. Regrettably, only 63% of patients in the chemoradiation arm of INT-0099 completed all three cycles of concurrent chemotherapy as planned. Ho et al. [20] compared the dose intensity and toxicity of CDDP administered weekly and every three weeks concurrently to 52 patients with locally advanced squamous head and neck cancer. They found that 100 mg/m^2^ CDDP every three weeks with RT was less well tolerated than 40 mg/m^2^ CDDP weekly or 80 mg/m^2^ CDDP every three weeks and resulted in fewer patients achieving a cumulative dose of >200 mg/m^2^. Therefore, the optimal CDDP dose in CCRT regimes for NPC warrants further exploration.

The dose intensity of CDDP is an important prognostic factor in NPC patients receiving CCRT [21]–[22]. Using pooled data from three prospective trials (in which most patients were treated with conventional RT), Loong et al. [21] explored the prognostic significance of the total CDDP dose delivered during CCRT and reported that patients with stage II or III NPC who received more than five weeks of 40 mg/m^2^ CDDP weekly had significantly better OS than patients who received less than five weeks. In our study, a cumulative dose of ≥160 mg/m^2^ led to obviously better OS, although the difference was not significant (84.1% vs. 73.3%, *P* = 0.175); this may be related to the small number of patients who received a cumulative dose of <160 mg/m^2^. Additionally, the use of IMRT has improved the long-term outcome for patients with NPC, which may render detection of the survival benefit of a higher total CDDP dose difficult.

The toxicity profiles of both groups were similar. However, there was a slightly higher incidence of grade 3–4 hematological toxicity and oropharyngeal mucositis in the weekly CDDP group, although the difference was not significant. The main reasons for this observation may be the inadequate monitoring of blood counts and poor oral care in outpatients in the weekly CDDP group. It is worth noting that there was a lower incidence of grade 3–4 dermatitis, dysphagia, and gastrointestinal reactions in patients who received CDDP weekly even though they received a relatively high median cumulative dose.

To our knowledge, this is the first single-institution study to review the difference between CDDP administered weekly and every three weeks concurrently with IMRT in NPC. The principal limitation of this study is its retrospective nature. However, the clinical characteristics of the patients in the two groups were well-balanced, which may reduce potential selection bias. To derive a definitive conclusion, a randomized phase II study of CCRT with CDDP administered every three weeks versus weekly in patients with locally advanced NPC is ongoing in Korea. The findings of this study will aid in the standardization of CCRT regimens for NPC.

## Conclusions

IMRT in conjunction with concurrent CDDP chemotherapy administered weekly or every three weeks leads to similar long-term survival outcomes and acute toxicities in NPC regardless of whether the patient has early- or advanced-stage disease. The results of this study require further validation in prospective, multi-center controlled trials.
